# An Approach to Quantifying the Interaction between Behavioral and Transmission Clusters

**DOI:** 10.3390/v14040784

**Published:** 2022-04-10

**Authors:** Luisa Salazar-Vizcaya, Katharina Kusejko, Huldrych F. Günthard, Jürg Böni, Karin J. Metzner, Dominique L. Braun, Dunja Nicca, Enos Bernasconi, Alexandra Calmy, Katharine E. A. Darling, Gilles Wandeler, Roger D. Kouyos, Andri Rauch, the Swiss HIV Cohort Study

**Affiliations:** 1 Department of Infectious Diseases, Bern University Hospital Inselspital, University of Bern, 3010 Bern, Switzerland; gilles.wandeler@insel.ch (G.W.); andri.rauch@insel.ch (A.R.); 2 Department of Infectious Diseases and Hospital Epidemiology, University Hospital Zurich, 8091 Zurich, Switzerland; katharina.kusejko@usz.ch (K.K.); huldrych.guenthard@usz.ch (H.F.G.); karin.metzner@usz.ch (K.J.M.); dominique.braun@usz.ch (D.L.B.); roger.kouyos@uzh.ch (R.D.K.); 3 Institute of Medical Virology, University of Zurich, 8057 Zurich, Switzerland; juerg.boeni@usz.ch; 4 Institute of Nursing Science, University of Basel, 4056 Basel, Switzerland; dunja.nicca@unibas.ch; 5 Department of Public Health, Epidemiology, Biostatistics and Public Health Institute, University of Zurich, 8001 Zurich, Switzerland; 6 Division of Infectious Diseases, Ente Ospedaliero Cantonale, 6900 Lugano, Switzerland; enos.bernasconi@eoc.ch; 7 University of Southern Switzerland, 6928 Manno, Switzerland; 8 Division of Infectious Diseases, Geneva University Hospitals, 1205 Geneva, Switzerland; alexandra.calmy@hcuge.ch; 9 Infectious Diseases Service, Lausanne University Hospital (CHUV), 1011 Lausanne, Switzerland; katharine.darling@chuv.ch; 10 University of Lausanne, 1015 Lausanne, Switzerland

**Keywords:** clusters, sexual behavior, transmission networks, hepatitis C virus

## Abstract

We hypothesize that patterns of sexual behavior play a role in the conformation of transmission networks, i.e., the way you behave might influence whom you have sex with. If that was the case, behavioral grouping might in turn correlate with, and potentially predict transmission networking, e.g., proximity in a viral phylogeny. We rigorously present an intuitive approach to address this hypothesis by quantifying mapped interactions between groups defined by similarities in sexual behavior along a virus phylogeny while discussing power and sample size considerations. Data from the Swiss HIV Cohort Study on condom use and hepatitis C virus (HCV) sequences served as proof-of-concept. In this case, a strict inclusion criteria contrasting with low HCV prevalence hindered our possibilities to identify significant relationships. This manuscript serves as guide for studies aimed at characterizing interactions between behavioral patterns and transmission networks. Large transmission networks such as those of HIV or COVID-19 are prime candidates for applying this methodological approach.

## 1. Introduction

Sexual behavior has changed among men who have sex with men (MSM) over the last two decades. In particular, condom use might have been influenced by changes in recommendations related to emerging scientific knowledge, availability of social media and the widespread use of stimulating substances for sex [1,2]. Such behavioral changes demonstrated heterogeneous trends [3,4,5,6]. Groups defined by similar patterns of change in sexual behavior could reflect similar adaptation mechanisms to changes in information capable of influencing risk perception. For instance, the popularization of the U = U message (undetectable equals untransmissible) [7,8,9] preceded by the Swiss Statement [10] might have reduced condom use during sexual intercourse with partners of HIV-negative or unknown statuses only among some persons living with HIV. The probability that such changes in risk behavior occur may vary across individuals and over time. For example, we recently identified clusters of sexual behavior with distinct time trends in condom use during anal intercourse [6]. We hypothesize that these behavioral clusters may play a role in the conformation of viral transmission networks and vice versa. If this was the case, behavioral groups inferred from temporal changes in sexual practices might in turn help predict proximity in transmission networks.

Clear characterizations of the relationships between sexual behavior and transmission networks may help identify targets for an efficient, new generation of public health interventions. We propose an approach to quantify such relationships. It uses intersections between clusters of sexual behavior and clusters of sexual transmission to infer interactions amongst clusters of sexual behavior. Because associations between condom use and incident infections with hepatitis C virus (HCV) have been shown previously [11], here we investigated HCV infections among HIV-positive MSM for a proof-of-concept using behavioral and HCV sequence data from the Swiss HIV Cohort Study (SHCS).

## 2. Materials and Methods

### 2.1. The Swiss HIV Cohort Study

The SHCS [12] is a nationwide prospective cohort that has routinely collected behavioral, laboratory and clinical data from HIV-positive persons aged ≥ 16 years since 1988. The cohort records individual data at study entry and in bi-annual follow-up visits. We estimate that more than 80% of all MSM currently diagnosed with HIV in Switzerland are followed in the cohort [13,14].

This analysis involved two types of clusters previously inferred: behavioral clusters (*BCs*) and transmission clusters (*TCs*).

*BCs:* Behavioral clusters are groups of MSM inferred by an unsupervised machine learning algorithm informed with 18 years of self-reported condom use with non-steady partners (nsCAI) recorded by the SHCS. The algorithm aims at a classification that minimizes differences in nsCAI patterns within members of the same group while maximizing these differences between members of different groups. We termed the resulting hierarchically represented groups, behavioral clusters (*BCs*). The top four *BC* of this classification revealed evidently divergent trajectories of nsCAI, indicating behavioral heterogeneity among MSM in the SHCS. Further characteristics of these *BCs* have been described in detail before [6,15].

Because the hierarchical structure of the clustering allows for shallower grouping (which naturally results in larger clusters), we also studied the two top *BCs*. In order to cover the broader behavioral spectrum, the present study included an additional group, comprising MSM who have never reported nsCAI (*BC0*).

We referred to the resulting sets of behavioral clusters as the “full classification” (top 4 + *BC0*) and the “three clusters classification” (top 2 + *BC0*).

*TCs:* HCV transmission clusters originate in a maximum-likelihood phylogenetic tree of a segment of the NS5B region of the virus. The tree includes HCV infections diagnosed and sequenced within the SHCS, and strains circulating across Europe. The *TCs* are defined as the 11 found monophyletic trees with a bootstrap support value larger than 70% containing SHCS MSM sequences. *TCs* have been described in detail before [16].

### 2.2. Intersections: Mapping Behavior on Transmission

We mapped membership to one of the *BCs* into one of the *TCs*. In accordance with the inclusion criteria for the primary two studies [6,16], we considered MSM enrolled in the Swiss HIV Cohort Study [12] with HCV sequences located in HCV transmission clusters (*TCs*). In the case of nested clusters, mapping preferred the cluster corresponding to the most external node. Men included in the analyses also were required to have at least two years of follow-up with respect to semi-annual questionnaires of sexual behavior.

**Figure 1:**
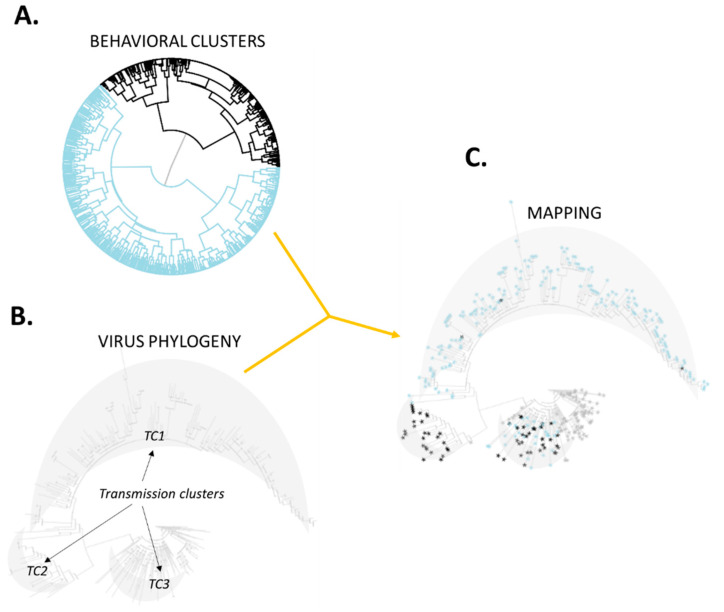
Hypothetical illustration of the mapping process. This example involves two behavioral clusters (panel (**A**); light blue and black) and three transmission clusters (*TC1*, *TC2* and *TC3* in panel (**B**)). The mapping shown in panel (**C**) suggests assortativity among members of the light blue behavioral cluster in transmission cluster *TC1* (containing only 2 members of the black behavioral cluster). Analogously, transmission cluster *TC2* is exclusively formed by members of the black behavioral cluster (an unlikely observation if mixing was at random). Members of both behavioral clusters distribute evenly along transmission cluster *TC3*.

Figure 1 illustrates this process with hypothetical versions of such pairs of clusters. It depicts 2 behavioral clusters (black and light blue in Figure 1A) and 3 transmission clusters contained in a virus phylogeny (Figure 1B). The subsequent mapping is illustrated by Figure 1C. In this hypothetical example, Figure 1C suggests assortativity between members of the light blue behavioral cluster along transmission cluster *TC1*.

This manuscript is concerned with the quantification of these patterns along the full virus phylogeny and the search for hints on potential associations between the two types of clustering (transmission versus sexual behavior).

### 2.3. Are MSM Who Share a Behavioral Cluster Likely to Also Share a Transmission Cluster?

To assess whether this was the case, we implemented a toy Monte Carlo analysis that iteratively shuffled transmission cluster membership at random. In each iteration, it compared the fraction of independent pairs of men who share a transmission cluster given that they share behavioral clusters, with the fraction of pairs of men who do not share transmission clusters given that they share behavioral clusters. Outcomes were odds ratios and their corresponding *p*-values.

### 2.4. Strength of Interaction between Behavioral Clusters

We intended to establish a measure of how prone members of behavioral clusters are to interact with each other over the whole set of HCV transmission clusters. For that purpose, let us assume that the distribution of behavioral cluster membership of sampled Swiss HCV sequences in each transmission cluster was generalizable, and mixing within HCV transmission clusters was homogeneous. Let us also assume interactions involving practices that could lead to HCV transmission occurring take place between MSM living in Switzerland. Given these assumptions, the probability over the whole HCV phylogeny that a member of behavioral cluster *BC_i_* interacts with a member of behavioral cluster *BC_j_* would be given by $\rho_{ij}=\frac{P_{ij}}{\sum_{i}P_{ij}}$, where $P_{ij}=\underset{k}{\sum }m_{BC_{i}}^{TC_{k}}f_{BC_{j}}^{TC_{k}}$ is our measure of interaction between these clusters, $m_{BC_{i}}^{TC_{k}}$ is the number of members of $BC_{i}$ in $TC_{k}$ and $f_{BC_{j}}^{TC_{k}}=\frac{\left(m_{BC_{j}}^{TC_{k}}-\delta_{i,j}\right)}{(\sum_{j’}m_{BC_{j’}}^{TC_{k}})-1}$ aims to reflect how common each behavioral cluster is in each transmission cluster. We used $\rho_{ij}$ to quantify the strength of mixing between behavioral clusters *BC_i_* and *BC_j_*.

### 2.5. Potential Influence of Behavioral Patterns on the HCV Phylogeny (Interaction Ratios)

We sought to identify over- or underrepresented mixing, as well as assortativity between pairs of behavioral clusters. We did this by estimating the ratio $r_{ij}$ between $\rho_{ij}$ and its expected value if *TCs* membership was independent of *BCs*, i.e., if behavioral clusters did not influence the conformation of HCV transmission clusters (or vice versa). If behavioral clusters did not influence HCV transmission clusters, the probability that a member of behavioral cluster *BC_i_* interacts with a member of behavioral cluster *BC_j_* would depend on the overall frequency of behavioral cluster *BC_j_* along the HCV phylogeny, i.e., ${\tilde{\rho }}_{ij}=\frac{(\sum_{k}m_{BC_{j}}^{TC_{k}})-\delta_{ij}}{(\sum_{k,j}m_{BC_{j}}^{TC_{k}})-1}$. The ratio is therefore given by $r_{ij}=\frac{\rho_{ij}}{{\tilde{\rho }}_{j}}$. This ratio approaches 1 when HCV transmission clustering is independent of behavioral clustering, while ratios above 1 (potential overrepresentation) and below 1 (potential underrepresentation) could reflect an effect of behavioral groups on HCV transmission clustering. For this analysis, we defined over- and underrepresentation as ratios above two and below 0.5, respectively.

### 2.6. Sample Size and Power Considerations

In order to assess our ability to demonstrate a relationship between behavioral and transmission clusters, we estimated the number of individuals necessary to achieve a significance level of 0.05 and a power of 70% for the toy Monte Carlo and the interaction ratios analyses.

### 2.7. Toy Monte Carlo Analysis

Experiments in this analysis were the number of independent pairs of men sharing *TC*. Independent pairs are those that cannot be inferred from other pairs. For example, if person *i* shares *TC* with person *j* and *k*, then *j* and *k* also share *TC*. While it adds up to 3 pairs sharing a cluster, only two would count as independent experiments.

### 2.8. The Interaction Ratios

These ratios were tested by assuming a stable structure of the intersection between the two types of clusters (i.e., the mapping). Therefore, that the ratio for a pair of *BC* required an increased number of members of these two clusters to reach a target level of significance, would also imply the need for increased numbers of members of all other clusters. This was in order to guarantee that all ratios resembled the original ones.

All algorithms were implemented in R running under Linux CentOS. Estimations relied on the R function prop.test and the R package pwr [17].

## 3. Results

**Figure 2:**
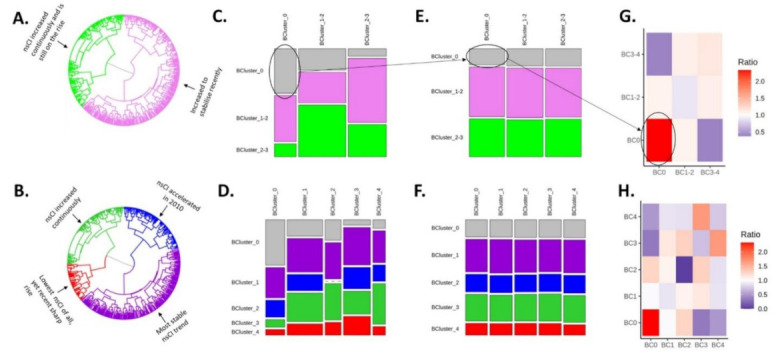
Interactions between behavioral clusters mediated by HCV transmission clusters. *Behavioral clusters diagrams* for: (**A**) The three clusters classification, and (**B**) the full classification. The cluster without nsCAI (*BC0*) is not shown since it does not emerge as an outcome of the clustering algorithm. *Mosaic plots of observed interactions* between behavioral clusters mediated by HCV transmission clusters for: (**C**) the three clusters classification and (**D**) the full classification. (Hint: vertical length of rectangles reflects the relative magnitude of the estimate). *Mosaic plots of expected interactions* between behavioral clusters mediated by HCV transmission clusters for: (**E**) the three clusters classification, and (**F**) the full classification. *Ratios between observed and expected mixing* amongst members of behavioral clusters for: (**G**) the three clusters classification and (**H**) the full classification. *Hint:* The grey rectangle (representing the interaction between members of *BC0*) in the observed diagram (**C**) is taller than that in the expected diagram (**E**). This indicates that members of *BC0* were more likely than expected to meet other members of the *BC0* in a transmission cluster. The corresponding cell in (**G**) is the most intense since it is also the strongest overrepresentation we found.

Size and nsCAI trajectories over time for the full classification and the Three clusters classification are briefly summarized in Figure 2A,B. Figure S1 shows the nsCAI trajectories over time for the no nil clusters in both classifications [6].

### 3.1. Intersections: Mapping Behavior on Transmission

We mapped membership to one of five *BCs* into each *TC*. Out of 4222 MSM with identified behavioral cluster membership, and 66 in the HCV phylogeny, 36 mapped to one of the *TCs*, i.e., had an HCV sequence located in a transmission cluster. Six members of cluster *BC0*, eleven of *BC1*, six of *BC2*, nine of *BC3* and four of *BC4* mapped to any of the *TCs*.

### 3.2. Are MSM Who Shared a Behavioral Cluster Likely to Also Share a Transmission Cluster?

Full classification: The odds ratio for sharing both behavioral and transmission clusters versus sharing behavioral but not transmission clusters after 10,000 iterations was 0.97 (*p*-value 0.61).

Sample size and power considerations: This analysis included 107 independent pairs of men sharing a transmission cluster and 433 not sharing a transmission cluster. To achieve a power of 70% at a level of significance of 0.05 for this ratio would require more than 98,000 men in each of these two groups.

### 3.3. Potential Influence of Behavioral Patterns on HCV Transmission Clustering

Figure 2C–F depict, for visual comparison, the observed (C and D) and expected (E and F) interactions between behavioral clusters mediated by HCV transmission clusters, for the full and the three clusters classifications, respectively. For instance, Figure 2C,E indicate that the estimated interaction between members of *BC0* was stronger than expected, suggesting that members of *BC0* are prone to share a transmission cluster. By contrast, the estimated interaction between members of clusters *BC0* and *BC3* was weaker than expected. Expected interactions constitute our null hypothesis and were assumed to only depend on the overall frequency of behavioral clusters along the HCV phylogeny, i.e., random grouping, so that interaction with larger clusters is more likely.

Figure 2G depicts the ratios between observed and expected interactions amongst members of the behavioral clusters along the HCV phylogeny for the three clusters classification. For instance, the largest resulting overrepresentation was found among members of *BC0* (2.3, *p*-value: 0.45), while the most prominent no nil underrepresentation was found to occur between members of *BC0* and members of *BC3-4* (0.37, *p*-value: 0.54). Analogously, Figure 2H depicts the respective ratios for the full classification. This ratio was also maximal for interactions among members of *BC0*, while we recorded no interactions among members of *BC2*.

### 3.4. Sample Size and Power Considerations

We estimated that overrepresentations and underrepresentations comparable to the strongest found in this proof-of concept could require three and four times as many mapped men (108 and 144 versus 36 available), respectively, to achieve a power of 70% at a level of significance of 0.05.

## 4. Discussion

We present an intuitive approach for quantifying associations between independently obtained behavioral and transmission clusters, which could help understand whether and how behavioral patterns relate to transmission networks. We used behavioral clusters based on condom use with non-steady partners and incidents of hepatitis C virus infections for proof-of-concept. Lack of power hindered our possibilities of obtaining conclusive outcomes for this proof-of-concept. This manuscript intends to serve as a guide for further, powered studies aimed at characterizing such associations.

This is the first study to quantify associations between clusters of sexual behavior and clusters of transmission derived from a viral phylogeny. We did this based on a simple, reproducible framework suitable for analogous and more general studies. Phylogenetic information bigger than that available for our proof-of-concept is, however, necessary to reach conclusive results (e.g., on a more prevalent, densely sampled infection, such as HIV or COVID-19).

The prevalence of HCV infection in the SHCS is low [18], and international transmission prominent [16]. We were therefore limited to small numbers, underscoring the need for larger transmission networks, as they would enable dense mapping. Our proof-of-concept relied on HCV infections, which resulted in a low-density mapping on the behavioral clusters, a risk that arises from analyzing a setting with a small population. While the HCV subtype 1a phylogenetic tree we used reached a sampling proportion above 60% (note that 60 sequences potentially relevant for our proof-of-concept were located in well-defined transmission clusters) [16], only 36 patients met the criteria to be included in this comparison.

Grounding an analysis of this nature on HCV among MSM in a geographical region with larger, denser transmission networks could reach the needed power and carry key advantages. Firstly, increased HCV transmission and changes in sexual behavior occurred simultaneously during the last years. Secondly, behavioral clusters depicted behavior after HIV diagnosis, and HCV infections in MSM have been most often observed to occur after HIV infection. Thirdly, the incidence of HCV has been on the rise among MSM in industrialized countries since the beginning of the 2000s [19,20,21], with recent signs of decline attributed to the scale-up of direct-acting antivirals [22,23].

That viral proximity and similar sexual behavior could be related has been hinted by recent studies on drivers of HIV transmission among MSM in Switzerland [24,25]. Kusejko and co-authors found that condomless sex was more common among HIV phylogenetic neighbors of MSM who did not use condoms [25]. Of note, our study does not rule out the possibility that HCV transmission networks reflect mixing between persons with high and low sexual risk behaviors, rather than mixing between persons with similar behavioral patterns. The HCV phylogeny could, for instance, reflect episodic exposures to transmission [26,27,28]. Such episodic transmission patterns have been shown to account for many HIV transmissions among MSM in London [27].

Relationships between behavior and transmission patterns may be mediated and at least partially explained by social networking through the exchange of information. The authors of a literature review of MSM networks research studies concluded that norms, attitudes and levels of exposure to HIV transmission were similar among members of the same social network [29]. Other studies involving behavior and transmission focused on behavior as a predictor of seroconversion. For instance, a study in Amsterdam identified three typical trajectories of anal intercourse with occasional partners among MSM over their life’s course and assessed the risk of HIV seroconversion across trajectories [30].

In summary, this manuscript presents a methodological approach to assess whether and how records on (sexual) behavior could aid the inference of (sexual) transmission networking. The characterization of such relationships could in turn inform approximations on transmission networking in the absence of genetic data. Large transmission networks such as those of HIV or COVID-19 are prime candidates for applying this methodological approach.

## Data Availability

The individual level datasets generated or analyzed during the current study do not fulfill the requirements for open data access: (1) The SHCS informed consent states that sharing data outside the SHCS network is only permitted for specific studies on HIV infection and its complications, and to researchers who have signed an agreement detailing the use of the data and biological samples; and (2) the data are too dense and comprehensive to preserve patient privacy in persons living with HIV. According to the Swiss law, data cannot be shared if data subjects have not agreed, or data are too sensitive to share. Investigators with a request for selected data should send a proposal to the respective SHCS address (www.shcs.ch/contact, accessed on 5 April 2022). The provision of data will be considered by the Scientific Board of the SHCS and the study team and is subject to Swiss legal and ethical regulations, and is outlined in a material and data transfer agreement.

## Supplementary Materials

The following supporting information can be downloaded at: https://www.mdpi.com/article/10.3390/v14040784/s1, Figure S1: Trends in condomless anal intercourse with non-steady partners among MSM across behavioral clusters [6]. Behavioral cluster *BC0* comprises only patients without reported nsCAI.
