# Supplementary material for: An Approach to Quantifying the Interaction between Behavioral and Transmission Clusters
Source: Viruses. 2022 Apr 10;14(4):784. doi: 10.3390/v14040784 (PMC9032082; doi:10.3390/v14040784)

An approach to quantifying the interaction between behavioural and transmission clusters

Luisa Salazar-Vizcaya, *et.al*

Figure S1. Trends in condomless anal intercourse with non-steady partners among MSM across behavioral clusters [6]. Behavioral cluster BC0 comprises only patients without reported nsCAI.

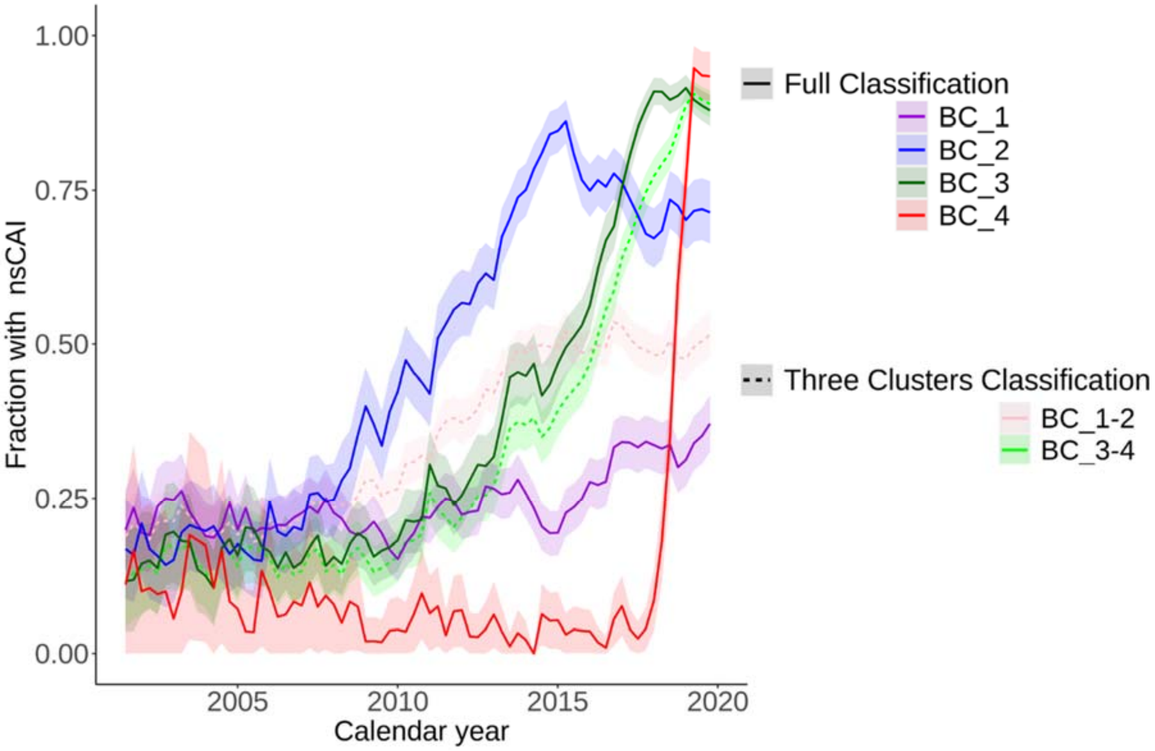

Supplement: Supplementary file 1 [file viruses-14-00784-s001.zip › viruses-1631158-supplementary.pdf]
